# Caregiver insights on challenges and needs in fecal incontinence products: a mixed methods study

**DOI:** 10.3389/fpubh.2025.1453244

**Published:** 2025-06-09

**Authors:** Lanlan Yu, Fengming Hao, Jie Li, Yingjie Hu, Fei Xiong, Ling Chen, Wenzhi Cai

**Affiliations:** ^1^Department of Nursing, Shenzhen Hospital, Southern Medical University, Shenzhen, China; ^2^School of Nursing, Southern Medical University, Guangzhou, China; ^3^ICU Department, Shenzhen Hospital, Southern Medical University, Shenzhen, China

**Keywords:** fecal incontinence, bedridden persons, caregivers, medical devices, incontinence-associated dermatitis, health policy

## Abstract

**Aims:**

This study explores the real–world use and challenges of fecal incontinence (FI) collection products—both absorbent items (pads, diapers) and dedicated fecal-collection devices with adhesive fixators—among long-term, bed-bound hospital patients, while also considering broader public-health implications. It seeks to identify barriers to optimal product use and to offer recommendations for improving incontinence management outcomes.

**Background:**

Effective FI management is essential to patient wellbeing and to preventing healthcare-associated infections (HAIs). Although multiple FI collection products are available, their use in long-term hospital settings remains suboptimal, largely because of caregiver training gaps and limited resources.

**Methods:**

This mixed-methods study used an explanatory sequential design. Quantitative data were gathered through online and paper-based surveys administered to caregivers in three hospitals (*n* = 318). These data were supplemented by qualitative interviews (*n* = 24) that provided deeper insight into the challenges identified. We performed descriptive and inferential statistical analyses, including logistic regression, and carried out a thematic analysis of interview transcripts to clarify the factors influencing product choice and the related public-health implications.

**Results:**

Product choice was shaped by distinct factors across caregiver groups. For family caregivers, household income (OR = 2.380) and living arrangement (OR = 0.344) were major determinants. Among nursing assistants, prior training (OR = 8.817) strongly affected selection. For nurses, incontinence-associated dermatitis training (OR = 3.344) and work environment (OR = 3.304) were critical. Qualitative interviews highlighted mismatches between available products and actual needs, emphasizing the importance of reforming procurement channels, raising awareness, and tailoring caregiver education.

**Conclusions:**

Disparities in FI product use stem mainly from economic constraints, training gaps, and limited awareness. Enhancing caregiver training, streamlining product distribution, and broadening insurance support could strengthen FI management and reduce HAIs. Although the findings offer useful guidance for policy and practice, their generalizability is limited by the single geographic setting and reliance on self-reported data. Future studies should examine diverse institutional contexts to validate and extend these results.

## 1 Introduction

Fecal incontinence (FI)—the involuntary loss of bowel control—affects people across the life span (1). Its prevalence in the general population is estimated at 2%−20.7%, but rates are substantially higher in specific groups, particularly among long-term, bed-bound hospital patients, of whom 16%−20% are affected (1–3). Beyond its physical burden, FI has significant public-health implications because it precipitates psychological and social distress—embarrassment, anxiety, and a reduced quality of life (4). Prolonged exposure to fecal matter also increases the risk of skin injury, such as incontinence-associated dermatitis (IAD), and of infections, including healthcare-associated infections (HAIs), which are a major concern in hospitals (5, 6).

Managing FI effectively is therefore critical not only for patient wellbeing but also for curbing HAIs, which impose a considerable burden on healthcare systems (7). Appropriate care can reduce cross-contamination and interrupt infection transmission, thereby improving both individual outcomes and public health (7, 8). Management typically combines medical, physical, and psychosocial strategies (8). In hospital settings, a central component is the use of FI-collection products that range from absorbent pads and diapers—originally designed for urinary incontinence but often used for FI—to dedicated bag-like devices with adhesive fixators (9, 10). In many regions, patients with comparable conditions may be transferred to nursing homes or long-term-care facilities; in our locality, however, they often remain hospitalized for extended periods.

Within long-term wards, caregivers—both healthcare professionals and family members—play a pivotal role in FI management (11). Professional caregivers such as nurses and nursing assistants must juggle FI care with numerous other duties amid time and resource constraints (12). Family caregivers, who usually lack formal medical training, may face limited knowledge, variable insurance coverage, and out-of-pocket costs that lead to suboptimal product selection (13). National health insurance partially or fully covers many FI products, but the level of coverage varies by patient plan. When the supplied items are insufficient or inappropriate, families often pay out-of-pocket to supplement supplies, and formal IAD assessment and management protocols are typically initiated only after signs of skin damage emerge.

Suboptimal FI management compromises patient comfort and outcomes, increases workload for healthcare staff, and heightens the risk of infection spread within hospitals (6, 14, 15). These shortcomings highlight the need for targeted interventions, including enhanced caregiver training, more efficient product-distribution systems, and policies that guarantee equitable access to advanced FI products. While disposable incontinence products have been examined in community and acute-care settings (13, 16), few studies have focused on long-term hospital wards, especially from the perspectives of multiple caregiver groups. Because effective FI management is crucial for preventing HAIs, understanding real-world product use, caregiver challenges, and factors that shape product choice is essential.

Accordingly, this study investigates how caregivers—nurses, nursing assistants, and family members—use and perceive FI-collection products for long-term, bed-bound hospital patients. By pinpointing barriers and unmet needs, we aim to inform public-health strategies that foster better FI management, lower HAI incidence, improve patient outcomes, and ease pressure on the healthcare system.

## 2 Methods

### 2.1 Study design

This research adopted a sequential explanatory mixed-methods design (17), integrating quantitative and qualitative approaches to provide breadth and depth in understanding real-world use of FI-collection products among bed-bound patients in long-term hospital settings.

#### 2.1.1 Phase 1 (October 2022–March 2023)

A cross-sectional survey assessed clinical use of FI-collection products, focusing on the prevalence of different product types and factors influencing caregivers' choices.

#### 2.1.2 Phase 2 (March 2023–May 2023)

**Figure 1:**
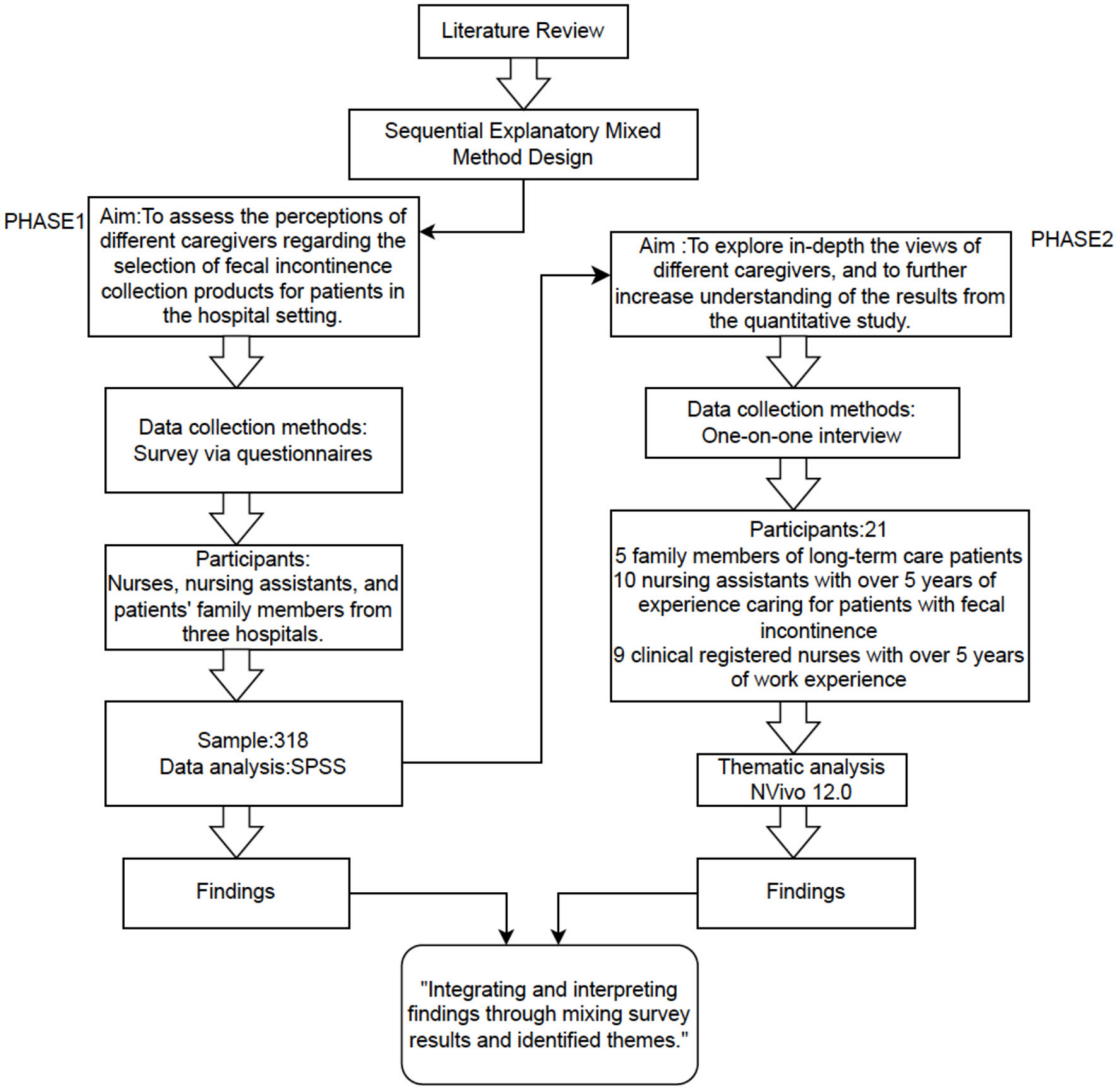
The constituent components of the mixed-methods approach conceptual framework.

Semi-structured interviews were conducted to elaborate on and contextualize the quantitative findings (Figure 1).

This integrative approach allowed qualitative themes to clarify the initial quantitative results, thereby strengthening the study's credibility and completeness. The quantitative portion followed the STROBE guidelines (18) (Appendix 1), whereas the qualitative portion adhered to the COREQ checklist (19) (Appendix 2).

### 2.2 Quantitative methods

#### 2.2.1 Setting and procedures

In Phase 1, we used convenience sampling to conduct a cross-sectional survey in three hospitals that care for long-term, bed-bound patients with FI. Eligible participants—nurses, nursing assistants, and family caregivers—received either an online questionnaire link (via WeChat) or a paper version if they preferred a non-digital format. Study objectives and assurances of anonymity were explained, and written informed consent was obtained.

Based on published guidance (20), the recommended sample size for a cross-sectional study is 5–10 times the number of independent variables. With 28 independent variables, the minimum sample size was 175 and the maximum 350 after adjusting for an anticipated 20% non-response rate [(28 × 5)/(1–0.2) to (28 × 10)/(1–0.2)].

Inclusion criteria were: (1) ≥18 years of age, (2) providing direct care to a bed-bound FI patient for ≥3 months, and (3) voluntary participation. A standardized questionnaire was used across all three hospitals without modification to ensure consistency.

#### 2.2.2 Data collection

The survey instrument was developed from a comprehensive literature review and refined through several rounds of expert consultation to ensure content validity. Key items captured demographic characteristics, product types used, and training or awareness related to FI management.

Data were collected from October 2022 to March 2023. Of 331 questionnaires returned, 13 were excluded because of incomplete demographic information, leaving 318 valid responses. Sex, age, marital status, education level, and other sociodemographic factors were recorded.

#### 2.2.3 Statistical analysis

Analyses were performed with IBM SPSS Statistics, version 26.0. Descriptive statistics summarized participant characteristics and product use; chi-square tests assessed associations between caregiver type and product choice. Binary and multivariate logistic regression examined relationships between the dependent variable (type of FI-product used) and independent variables—sex, age, education, household income, caregiver identity (family member, nursing assistant, nurse), long-term cohabitation with the patient, and specialized training (e.g., IAD training for nurses). Throughout, “sex” refers to biological classification rather than self-identified gender.

### 2.3 Qualitative methods

#### 2.3.1 Recruitment and participants

To supplement the quantitative findings, we conducted in-depth, semi-structured interviews with 24 participants-−9 registered nurses, 10 nursing assistants, and 5 family caregivers—selected through purposive sampling in collaboration with hospital administrators. Eligibility criteria were: (1) caring for FI patients ≥30 h/week for at least 6 months, (2) ≥18 years of age, and (3) fluency in Mandarin Chinese. Sampling continued until data saturation was achieved.

#### 2.3.2 Data collection

The primary investigator (LLY), an experienced nurse and qualitative researcher, conducted face-to-face interviews in private conference rooms within each hospital. Because LLY shared a nursing background with many participants—an advantage for building rapport but a possible source of pre-conceptions—she maintained reflexive memos after every interview and regularly debriefed with two co-authors to challenge emerging interpretations and minimize disciplinary bias. Each interview lasted between 31 and 97 min, was audio-recorded, and transcribed verbatim. The semi-structured interview guide covered three broad themes: (1) perceptions of using FI collection products, (2) facilitators and barriers to clinical application, and (3) current challenges in FI management. To protect participants' autonomy and wellbeing, they were informed of their right to withdraw from the study at any point without any repercussions and to refuse to answer any questions that made them uncomfortable. Additionally, the interviewer remained mindful of the emotional and physical burden often associated with caregiving, offering participants the option to pause or take breaks as needed, and approached sensitive topics in a non-judgmental manner. Field notes were taken concurrently to capture non-verbal cues and contextual details. We continued interviewing until no new themes emerged, indicating data saturation.

#### 2.3.3 Data analysis

Transcripts were analyzed using Braun and Clarke's (40) inductive thematic analysis approach, which involves six steps: (1) data familiarization, (2) initial code generation, (3) theme search, (4) theme review, (5) theme naming, and (6) final reporting. Two researchers (LLY and JL) independently coded the transcripts and discussed initial themes with the broader research team (WZC, LC, FMH, YJH, and FX) to ensure consensus and trustworthiness. Discrepancies were resolved through group discussion. This collaborative approach enriched theme development by incorporating diverse professional perspectives, thereby enhancing the depth and reliability of our qualitative findings.

### 2.4 Ethics considerations

Participation was voluntary; respondents could decline any question. Written informed consent was obtained before survey completion and interviews. Ethical approval was granted by the institutional ethics committee on 13 October 2022 (approval no. NYSZYYEC20220033).

## 3 Results

### 3.1 Quantitative findings

A total of 331 caregivers completed the survey; after excluding 13 incomplete or invalid questionnaires, 318 valid responses remained, for an overall response rate of 96.1 %. The final sample included 115 family caregivers, 73 nursing assistants, and 130 registered nurses—all of whom cared for long-term, bed-bound patients with fecal incontinence (FI) in the three participating hospitals.

#### 3.1.1 Demographic data

*Family caregivers (n* = *115)*. Most respondents were female (68/115, 59.1%), married (87/115, 75.7%), and employed (76/115, 66.1%). The majority were the patient's children (85/115, 73.9%), followed by spouses (20/115, 17.4%) and parents (10/115, 8.7%). Nearly all caregivers (110/115, 96.0%) reported that the patient had health-insurance coverage. With respect to FI products, 75 caregivers (65.2%) used standard absorbent items, whereas 40 (34.8%) opted for premium or branded absorbent products (Table 1).

**Table 1:** Demographic information of patients' family members in the quantitative survey (*N* = 115).

**Variable**	**n**	**%**
**Sex**		
Male	47	40.9
Female	68	59.1
**Age (years)**		
18–24	12	10.4
25–45	49	42.6
46–65	48	41.7
Over 65	6	5.2
**Educational level**		
Junior high school education or below	31	27
High school	38	33
College diploma	29	25.2
Undergraduate degree	17	14.8
**Marital status**		
Married	87	75.7
Unmarried	28	24.3
**Employment status**		
Employed	76	66.1
Unemployed	39	33.9
**Monthly household income**		
0–10,000	70	60.9
Over 10,000	45	39.1
**Relationship to the patient**		
Spouse	20	17.4
Children	85	73.9
Parents	10	8.7
**Long-term cohabitation with the patient**		
Yes	34	29.6
No	81	70.4
**Does the patient have medical insurance?**		
Yes	110	95.7
No	5	4.3
**Patient's awareness**		
Conscious	42	36.5
Consciousness disorder	73	63.5
**Absorbent product choice**		
Branded absorbent products	40	34.8
Generic absorbent products	75	65.2

*Nursing assistants (n* = *73)*. Most were female (61/73, 83.6%), and the largest age group was 46–55 years (51/73, 69.9%). Over half (41/73, 56.2%) had a middle-school education or less, and 46 (63.0%) had 1–5 years of caregiving experience; only 7 (9.6%) reported more than 10 years. More than half (38/73, 52.1%) had not received formal training in FI or incontinence care, and the majority (58/73, 79.5%) were from rural areas (Table 2).

**Table 2:** Demographic information of nursing assistants in the quantitative survey (*N* = 73).

**Variable**	***n***	**%**
**Sex**		
Male	61	83.6
Female	12	16.4
**Age (years)**		
18–35	11	15.1
36–55	51	69.9
Over 55	11	15.1
**Educational level**		
Junior high school education or below	41	56.2
High school	22	30.1
College diploma or above	10	13.1
**Working experience (years)**		
1–5	46	63
6–11	20	27.4
Over 11	7	9.6
**Training**		
Yes	35	47.9
No	38	52.1
**Areas of origin**		
Rural	58	79.5
Urban	15	20.5
**FI Collection product choice**		
Absorbent products	63	86.3
Internal or External FI collection products	10	13.7

*Registered nurses (n* = *130)*. Most were female (112/130, 86.2%) and between 25 and 45 years of age (109/130, 83.8%). More than half (83/130, 63.9%) had over 5 years of clinical experience. Notably, 95 nurses (73.1%) had worked in ICUs, yet only 30 (23.1%) had received specialized training in IAD. Educational attainment included 3 nurses (2.3%) with master's degrees, 100 (76.9%) with bachelor's degrees, and the remainder with associate degrees (Table 3).

**Table 3:** Demographic information of registered nurses in the quantitative survey (*N* = 130).

**Variable**	***n***	**%**
**Sex**		
Male	18	13.8
Female	112	86.2
**Age (years)**		
18–25	13	10
26–35	59	45.3
36–45	50	38.5
46–55	7	5.4
Over 55	1	0.8
**Educational level**		
College diploma	27	20.8
Undergraduate degree	100	76.9
Master's degree or above	3	2.3
**Working experience (years)**		
1–5	36	27.2
6–10	47	36.2
11–21	36	27.2
Over 21	11	8.5
**ICU working experience**		
Yes	35	26.5
No	95	73.1
**Specialist training in incontinence dermatitis**		
Yes	30	23.1
No	100	76.9
**FI collection product choice**		
Absorbent products	73	56.2
External FI collection products	45	34.6
Internal FI collection products	12	9.2

#### 3.1.2 Current use of FI collection products

##### 3.1.2.1 Family caregivers

**Figure 2:**
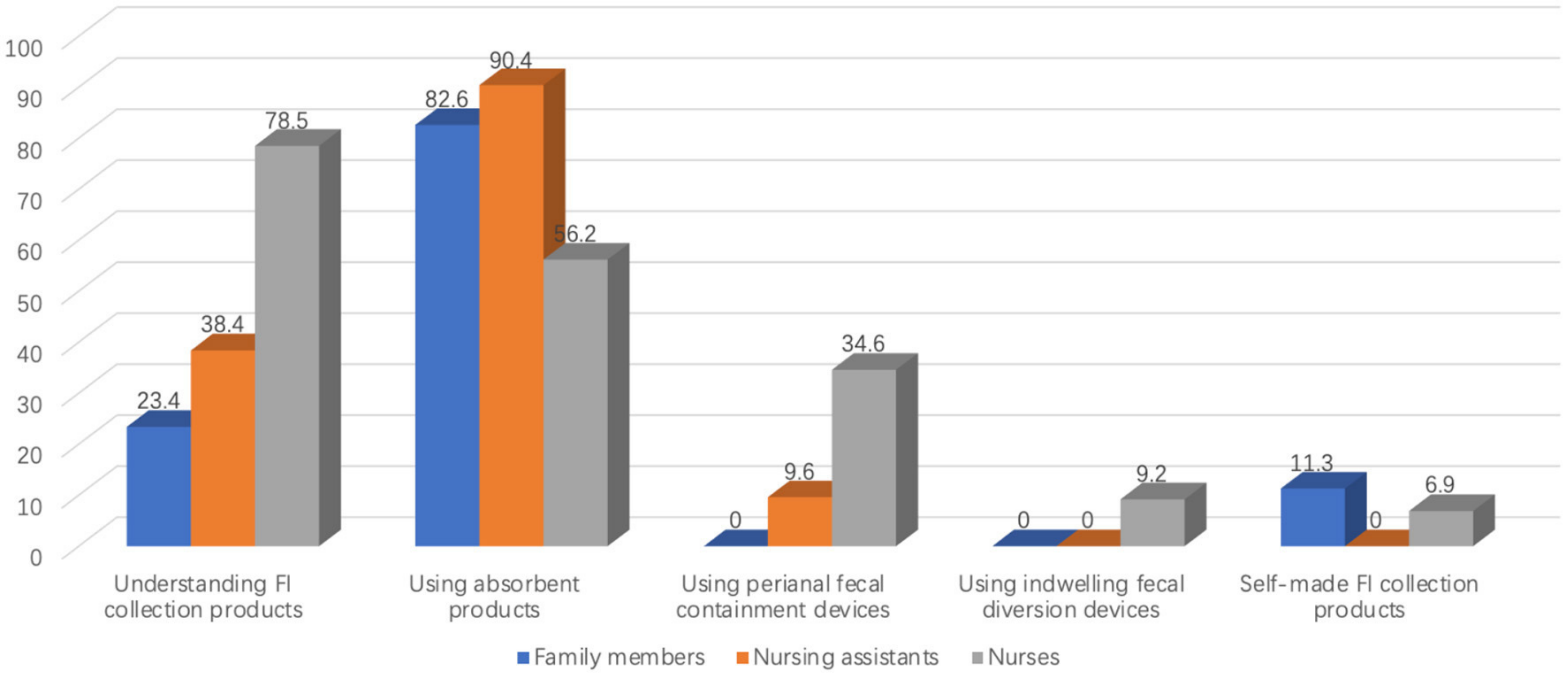
Usage of fecal incontinence collection products by different caregivers.

**Figure 3:**
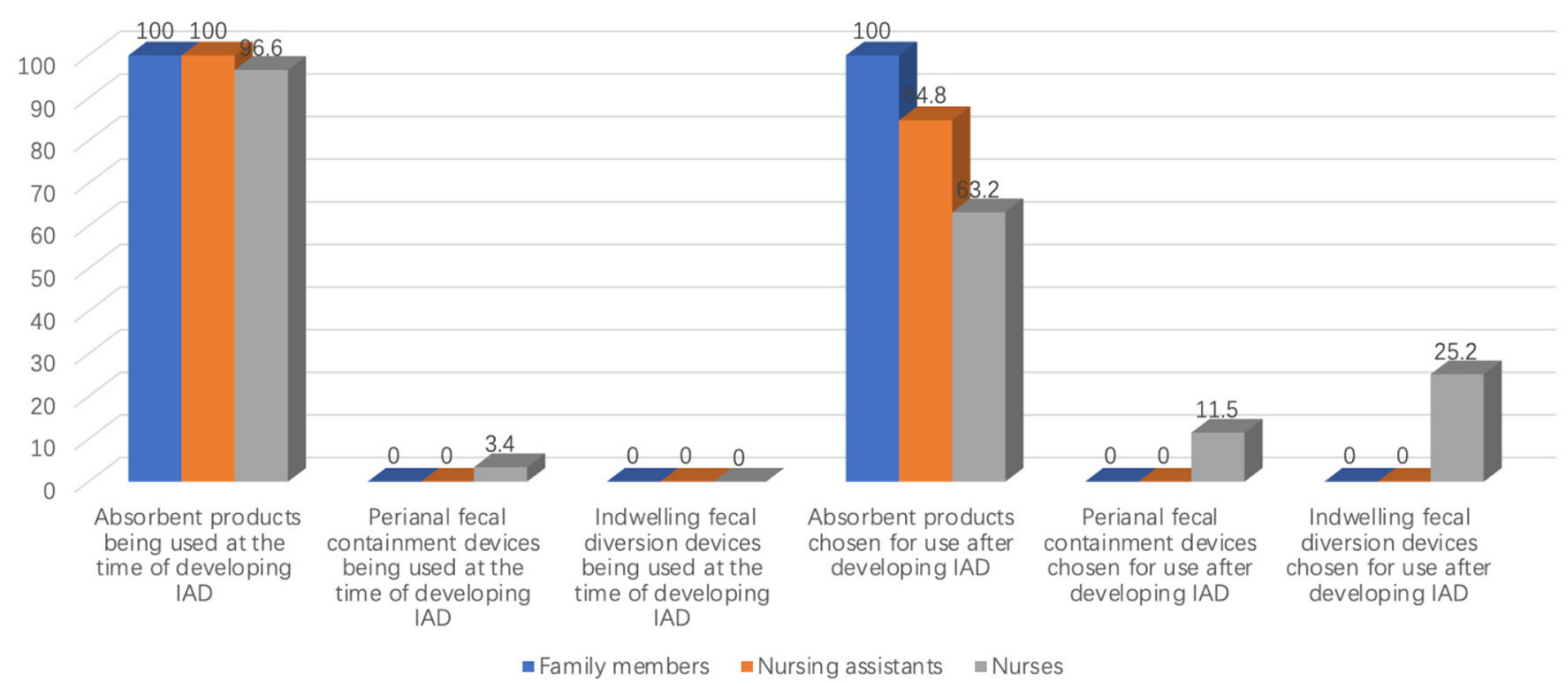
Usage of fecal incontinence collection products by different caregivers.

Only 27 of 115 caregivers (23.4%) were aware of FI-product options beyond standard absorbent pads. The vast majority relied on absorbent products (95/115, 82.6%); none reported using peri-anal or indwelling fecal-collection devices. A small subset (13/115, 11.3%) fashioned “homemade” FI-collection items (Figure 2). Among caregivers looking after patients with IAD (*n* = 27), all (27/27, 100%) continued to use absorbent products after IAD onset (Figure 3).

##### 3.1.2.2 Nursing assistants

Only 7 of 73 assistants (9.6%) were aware of peri-anal fecal-collection devices, and 3 (4.1 %) knew of indwelling diversion devices. In practice, 66 assistants (90.4%) used standard absorbent products; 7 (9.6%) had ever used a peri-anal device, and none had experience with an indwelling device (Figure 2). Among patients who developed IAD (*n* = 33), all assistants initially relied on absorbent products, and 28 (84.8%) continued to do so after IAD appeared (Figure 3).

##### 3.1.2.3 Registered nurses

Absorbent products were most frequently used (73/130, 56.2%), whereas 45 nurses (34.6 %) reported experience with peri-anal fecal-collection devices, and 12 (9.2%) had used indwelling devices (Figure 2). Of these 12 nurses, 3 had applied commercial FI-management kits, while the remainder improvised devices such as connecting a tracheostomy tube to a drainage bag. Among nurses who had managed IAD patients (*n* = 87), 10 (11.5%) eventually adopted indwelling devices (Figure 3).

#### 3.1.3 Factors influencing caregivers' product choices

##### 3.1.3.1 Family caregivers

Fisher's exact test revealed significant associations between family income, cohabitation status, patient awareness, and the choice of FI-collection products (*p* < 0.05; Table 4). Binary logistic regression confirmed that higher family income (OR = 2.380, 95% CI 1.036–5.464), long-term cohabitation with the patient (OR = 0.344, 95% CI 0.124–0.950), and patient awareness (OR = 2.616, 95 % CI 1.051–6.511) were each independently associated with product preference (Table 5).

**Table 4:** Association between family members' demographics and absorbent product choices (*N* = 115).

**Variables**	**Branded absorbent products**	**Generic absorbent products**	**χ^2^**	***p* value**
**Monthly household income**			4.603	0.045
0–10,000	51 (72.9)	19 (27.1)		
Over 10,000	24 (53.3)	21 (46.7)		
**Long-term cohabitation with the patient**			6.248	0.018
Yes	47 (58.0)	34 (42.0)		
No	28(82.4)	6 (17.6)		
**Patient's awareness**			5.201	0.026
Conscious	33(78.6)	9(21.4)		
Consciousness disorder	42(57.5)	31(42.5)		

χ^2^, chi-square value; *p* value, two-tailed statistical characteristic.

**Table 5:** Binary logistic regression analysis of the factors associated with the choice of absorbent products by the relatives of patients (*N* = 115).

**Variable**	**β**	**SE**	**OR (95% CI)**	***p* value**
Monthly household income	0.867	0.424	2.380 (1.036, 5.464)	0.041
Long-term cohabitation with the patient	−1.068	0.519	0.344 (0.124, 0.950 )	0.040
Patient's awareness	0.962	0.465	2.616 (1.051, 6.511 )	0.039

β, Beta coefficient; SE, Standard error; OR, odds ratio; 95% CI, 95% confidence interval.

##### 3.1.3.2 Nursing assistants

Education level and formal training were significantly related to product selection (*p* < 0.05; Table 6). Logistic-regression analysis showed that holding a college degree (OR = 15.511, 95% CI 2.020–119.120) and having received formal training (OR = 8.817, 95% CI 1.347–57.348) greatly increased the likelihood of choosing external or indwelling devices rather than absorbent products (Table 7).

**Table 6:** Association between nursing assistants' preferences and selection of FI collection products (*N* = 73).

**Variables**	**Absorbent product**	**Internal or External FI collection products**	**χ^2^**	***p* value**
**Educational level**			7.264	0.026
Junior high school education or below	38 (92.7)	3 (7.3)		
High school	19 (86.4)	3 (13.6)		
College diploma or above	6 (60.0)	4 (40.0)		
**Training**			4.77	0.041
Yes	36 (94.7)	2 (5.3)		
No	27 (77.1)	8 (22.9)		

FI, Fecal incontinence; χ^2^, chi-square value; *p* value, two-tailed statistical characteristic.

**Table 7:** Binary logistic regression analysis of factors influencing the selection of various FI collection products by nursing assistants (*N* = 73).

**Variable**	**β**	**SE**	**OR (95% CI)**	***p* value**
College diploma or above	2.742	1.04	15.511 (2.020,119.120)	0.008
Training	2.177	0.959	8.817 (1.347,57.348)	0.023

FI, Fecal incontinence; β, Beta coefficient; SE, Standard error; OR, odds ratio; 95% CI, 95% confidence interval.

##### 3.1.3.3 Registered nurses

Chi-square tests indicated that sex, ICU experience, and IAD-specific training were each significantly associated with the type of FI product used (*p* < 0.05; Table 8). In multivariate logistic regression, IAD training was the strongest predictor of selecting an indwelling device instead of absorbent pads (OR = 5.800, 95% CI 1.436–23.431). Use of peri-anal or external devices was associated with sex (OR = 4.091, 95% CI 1.224–13.680), ICU experience (OR = 3.034, 95% CI 1.228–7.498), and IAD training (OR = 3.344, 95% CI 1.318–8.944) (Table 9).

**Table 8:** Association between nurses' preferences and selection of FI collection products (*N* = 130).

**Variable**	**Absorbent product**	**External FI collection products**	**Internal FI collection products**	**χ^2^**	***p* value**
**Sex**				6.894	0.032
Male	68 (60.7)	35 (31.3)	9 (8.0)		
Female	5 (27.8)	10 (55.6)	3 (16.7)		
**ICU working experience**					
Yes	60 (63.2)	28 (29.5)	7 (7.4)	7.103	0.029
No	13 (37.1)	17 (48.6)	5 (14.3)		
**Specialist training about IAD**				6.610	0.037
Yes	62 (62.0)	7 (7.0)	31 (31.0)		
No	11 (36.7)	5 (16.7)	14 (46.7)		

FI, Fecal incontinence; IAD, Incontinence-associated dermatitis; χ^2^, chi-square value; *p* value, two-tailed statistical characteristic.

**Table 9:** Multinomial logistic regression analysis of factors influencing the selection of various FI collection products by nurses (*N* = 130).

**Variable**		**β**	**SE**	**OR (95% CI)**	***p* value**
External FI collection products	Sex	1.409	0.616	4.091 (1.224,13.680)	0.022
	ICU working experience	1.11	0.462	3.034 (1.228,7.498)	0.016
	Specialist training about IAD	1.234	0.488	3.434 (1.318,8.944)	0.012
Internal FI collection products	Specialist training about IAD	1.758	0.712	5.800 (1.436,23.431)	0.014

Reference Category: Absorbent Products. FI, Fecal incontinence; IAD, Incontinence-associated dermatitis; β, Beta coefficient; SE, Standard error; OR, odds ratio; 95% CI, 95% confidence interval.

### 3.2 Qualitative findings

**Figure 4:**
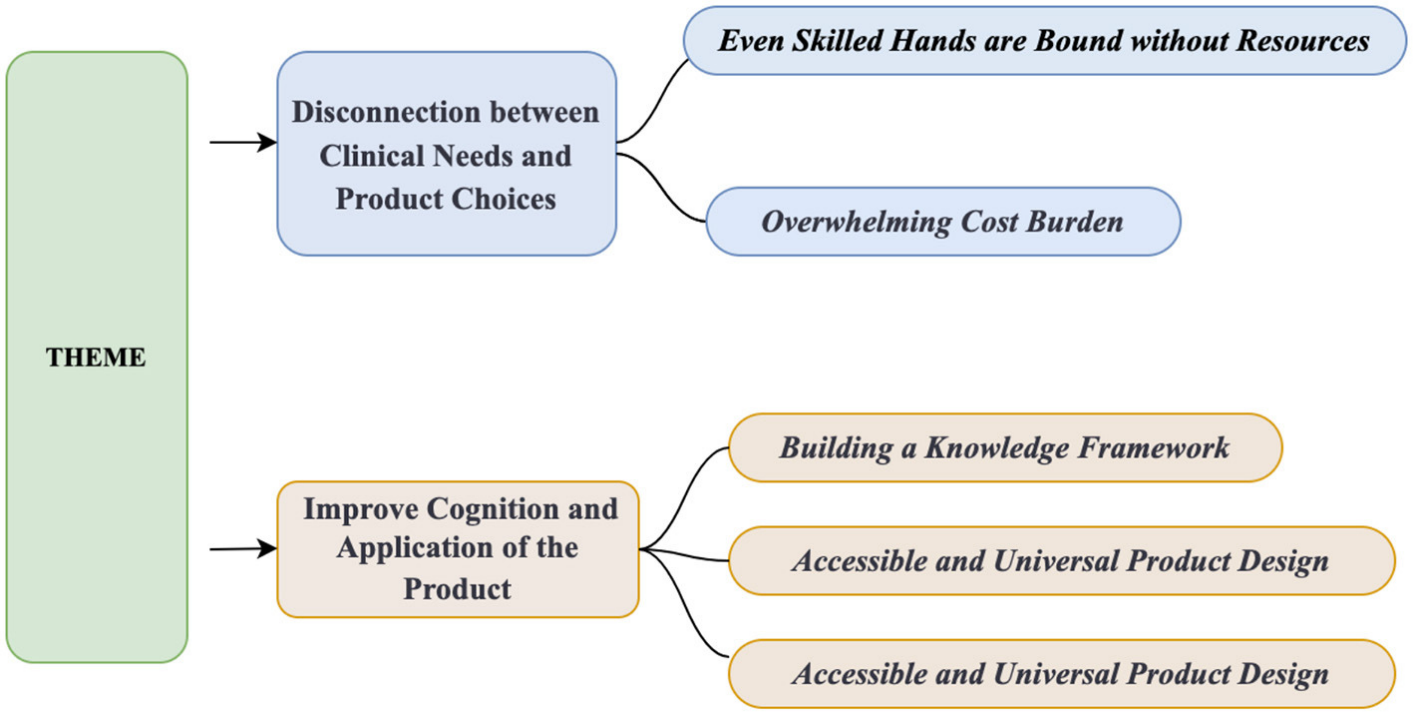
Main theme and subtheme in the qualitative data thematic analysis.

We conducted in-depth interviews with 24 participants: five family caregivers (two men and three women, aged 41–65 years), ten nursing assistants (all women, aged 37–58 years), and nine registered nurses (three men and six women, aged 28–50 years). Among the nurses, one was an ostomy specialist and another held a master's degree with 5 years of ICU experience. Table 10 summarizes participant characteristics. Two main themes and five sub-themes emerged (Figure 4).

**Table 10:** Characteristics of various caregiver participants (*n* = 24).

**No**.	**Caregivers**	**Gender**	**Age**	**Education**	**Experience, years**
1	Family member 1	W	61	Undergraduate degree	10
2	Family member 2	M	47	Undergraduate degree	3
3	Family member 3	M	65	College diploma	6
4	Family member 4	W	41	High school	1
5	Family member 5	W	50	High school	2
6	Nursing assistant 1	W	54	High school	21
7	Nursing assistant 2	W	56	Elementary school	10
8	Nursing assistant 3	W	48	Middle school	3
9	Nursing assistant 4	W	52	Elementary school	6
10	Nursing assistant 5	W	50	High school	11
11	Nursing assistant 6	W	56	Middle school	8
12	Nursing assistant 7	W	55	Middle school	9
13	Nursing assistant 8	W	57	Elementary school	14
14	Nursing assistant 9	W	51	Middle school	5
15	Nursing assistant 10	W	55	Middle school	11
16	Respiratory medical nurse 1	W	28	4-year bachelor	6
17	ICU nurse 2	W	34	Master	8
18	Neurology nurse 3	W	41	Undergraduate degree	21
19	Neurology nurse 4	W	50	College diploma	30
20	ICU nurse 5	M	35	4-year bachelor	12
21	Ostomy nurse 6	W	48	Undergraduate degree	28
22	Neurosurgery nurse 7	M	34	4-year bachelor	12
23	Gastroenterology nurse 8	W	44	Undergraduate degree	25
24	ICU nurse 9	M	32	4-year bachelor	9

#### 3.2.1 Main theme 1: disconnection between clinical needs and product choices

##### 3.2.1.1 Even skilled hands are bound without resources

One nurse invoked the Chinese idiom “hard to make bricks without straw” to describe FI-product use in the hospital: apart from absorbent pads, no other options were available. To improve patient care, she had previously improvised an FI-collection device herself. Another nurse added that patients with severe FI should have access to a wider range of products, yet in reality none exist; he therefore relies solely on incontinence pads, which adds to the workload and stress of caregiving.

“*I am aware of other FI collection products, such as FI management kits… Patients with different conditions have different needs for FI collection products, though almost every patient will use incontinence pads. I have the expertise, but when I want to switch products based on a patient's condition, I can only improvise using endotracheal tubes for fecal drainage, because there are no ostomy drainage products available for selection - alas, it feels like trying to make bricks without straw.” (Nurse 2)*

“*Once while rescuing another patient, one of my other patients happened to have an episode of FI, but I could only attend to the patient with the more severe condition, that is the one I was engaged in rescuing. As such, I simply did not have time to tend to the incontinent patient whose skin potentially remained soaked in feces for a prolonged period, quickly developing IAD… The pressure was immense.” (Nurse 6)*

Six nurses and one nursing assistant with 21 years of experience reported that the products they most often used or encountered were incontinence pads and diapers. Although they recognized the value of these absorbent items, they noted that the products sometimes fall short—particularly during episodes of liquid FI—because they do not provide adequate skin protection for patients.

“*Incontinence pads and diapers play an important role and reduce our burden for patients with routine FI. However, for patients with frequent liquid FI, their value lies only in protecting bed linens from fecal contamination rather than protecting patient skin; they may even aggravate skin damage.” (Nursing assistant 1)*

##### 3.2.1.2 Overwhelming cost burden

Participants emphasized the critical need for FI-collection devices to support patient care, noting that such products help resolve multiple caregiving challenges. Nevertheless, the mental strain and out-of-pocket expenses involved in obtaining these devices impose a substantial economic burden. For family members, cost was the predominant concern, whereas nurses and nursing assistants reported exhaustion and discouragement stemming from the physical and psychological demands of care.

“*I have cared for my mother for 6 years. Incontinence pads helped resolve the frequent sheet changes…I do not mind the hardship…Our family spends about 300 RMB monthly on pads and diapers…Even if more advanced products existed, regardless of quality, I would not buy them as I cannot afford the cost…” (Family member 3)*.

“*Frequently changing incontinence pads is physically exhausting for me on top of other duties, creating mental strain. Frankly, I dread FI patients in my ward (laughs)…I already have severe back injuries.” (Nurse 7)*

“*…the main issue is being unable to rest…after working like this for days, people become absent-minded…but this is my job, I can only persevere.” (Nursing assistant 3)*

#### 3.2.2 Main theme 2: improve cognition and application of the product

##### 3.2.2.1 Building a knowledge framework

At least two family caregivers proposed developing an app devoted to FI-care education, including information on product features and appropriate use scenarios. Such an app would allow caregivers to access guidance at any time, particularly after patients are discharged. They believed that ready access to this information could reduce avoidable rehospitalizations among incontinent patients and, in turn, lessen the burden on families.

“*I believe developing educational software such as a mobile app dedicated to FI would be helpful. The required knowledge and products could be provided through the app… We also have the capacity to learn - it would reduce avoidable rehospitalizations, benefiting us and you as well.” (Family member 2)*

Interviewed nursing assistants felt that a lecture-based format was ill-suited to acquiring caregiving knowledge. They recommended that hospitals create nursing-expertise consultation centers and distribute comprehensive informational brochures—highlighting product images—which they believed would be more helpful.

“*Lectures are ineffective for us; our main learning is through experience sharing with colleagues, though some are reluctant to share all their expertise. I can consult nurses but they are busy and sometimes unsure about my questions, with some being condescending. Having accessible experts for egalitarian consultation would be ideal, as would image-focused brochures.” (Nursing assistant 6)*

Interviewed nurses emphasized that caring for patients with FI—including the management of collection products—cannot rest solely with nursing staff. They stressed the need for close collaboration among nurses, physicians, and nutritionists and called for the formation of multidisciplinary expert teams. The nurses also expressed concern that such collaboration is currently lacking.

“*I want to emphasize that managing FI is not solely a nursing issue; physicians and nutritionists play equally important roles. The priority for FI patients is preventing IAD, yet physicians only pay attention after its onset, and nutritionists rarely proactively monitor patients. With equal prioritization from all three disciplines, establishing expert teams for early intervention when FI begins would certainly decrease IAD. However, I feel physicians and nutritionists lack the requisite knowledge and awareness, making this very difficult.” (Nurse 9)*

##### 3.2.2.2 Accessible and universal product design

Across all three caregiver groups, participants stressed that FI-collection products should be convenient and user-friendly for patients and caregivers alike—a priority that becomes even more critical for older caregivers with multiple comorbidities. They agreed that products capable of promoting patient wellbeing while easing caregiver burden would be widely embraced. Caregivers further emphasized that devices suited to different user groups—nurses, aides, and family members—and accessible regardless of age, physical ability, or health status are not only practical but also enhance caregiving efficiency for everyone.

“*I am 65 years old and have many illnesses. My children have to go to work… Taking care of her toileting needs is very strenuous for me. Even just changing a diaper requires help from a nurse, but once we are discharged home, it becomes a challenge… I certainly hope for more convenient products, especially those suitable for the older adult, which would also be better for my spouse.” (Family Caregiver 3)*

“*Ordinary patients' diapers are easy to change (laughs). It might be difficult at first, but with time, it becomes routine. However, when the patient is very overweight, it is still quite challenging. I am small and not very strong, so I hope there are products that can address the needs of 'overweight patients'…” (Caregiver 4)*

“*Adhesive pads for anal incontinence are definitely friendly for patients with loose stools, but they have limitations for overweight patients and females (laughs). They are very difficult to apply, and even when applied, they fall off quickly because they do not fit well.” (Nurse 2)*

##### 3.2.2.3 The trend toward smart products

Respondents repeatedly highlighted the need for intelligent FI-collection products. Family caregivers and aides prioritized devices capable of automatically cleansing perianal skin, whereas nurses emphasized additional features such as alarm systems, automated feces removal, and automatic data storage.

“*Nowadays, everything is smart, even robots. Fecal incontinence (collection) products can also be intelligent, for example, helping my mother clean her perianal skin. This would save me the cost of hiring a caregiver.” (Family Caregiver 2)*

“*I really hope for the production of products that can automatically help clean patients, not only cleaning the 'buttocks' (perianal skin) but also the whole body, as bathing bedridden patients is also very challenging (laughs).” (Aide 1)*

“*If the product could issue alarms and even automatically handle feces, and store data, I could collect data on the timing of incontinence-related dermatitis. But this is a wishful thinking (laughs).” (Nurse 5)*

## 4 Discussion

This multicenter study underscores the substantial challenges of managing FI in long-term hospital settings, notably the limited range of products and the underuse of advanced devices. Although newer technologies—such as peri-anal or indwelling fecal-management systems—are available, absorbent products remain the predominant choice across all caregiver groups, even when IAD is present. These findings corroborate earlier work showing that high-quality absorbent pads can retain excess moisture and heighten IAD risk, particularly in bed-bound older adults with frequent liquid stool (21–23). By contrast, when stool is non-liquid, saturation indicators have limited value for assessing performance or signaling timely intervention. Effective FI-management strategies should therefore combine absorbent products, fecal-diversion systems, and structured skin-care protocols—tailored to clinical context, including the use of indwelling devices or fecal bags for liquid stool incontinence (9, 10, 24).

Financial constraints and limited training compound these issues. Family caregivers frequently cited cost as a barrier to adopting advanced products, echoing research that economic factors strongly influence caregiving decisions (25). Conversely, nursing assistants and nurses with formal training were more willing to consider peri-anal or indwelling devices, yet actual use remained low because of supply-chain gaps and the perceived complexity of advanced products. Improvised solutions—such as large-diameter urinary catheters repurposed as rectal tubes—highlight the disconnect between clinical needs and the paucity of standardized devices (24). Such practices jeopardize patient safety and point to an urgent need for policy reforms that guarantee hospitals a broader array of regulated FI products.

Knowledge gaps further hinder optimal care. Many caregivers rely on absorbent pads without medical guidance (26). This deficit is particularly pronounced among family caregivers and nursing assistants: product choice correlates with education level and formal training. Trained nursing assistants more often select indwelling or external devices, and nurses—especially those with ICU experience—are strongly influenced by IAD-specific training, a finding that contrasts with earlier reports (27). Public health policies should prioritize comprehensive training programs for caregivers and resource allocation to ensure that both hospital and community caregivers are equipped with the skills necessary to manage FI effectively. This includes providing free nursing resources and product support for family caregivers, particularly in low-income households, to prevent the health risks associated with the use of ineffective products due to economic constraints.

Interview data indicate that practice gaps stem primarily from missing structured education rather than product cost. Caregivers prefer simple resources—mobile apps, illustrated guides, and expert hotlines—to help them choose appropriate products, follow skin-care protocols, and detect early IAD. Targeted training is associated with improved product use, reduced caregiver burden, and lower IAD rates (28–30). Brief, scalable modules should therefore be embedded in discharge teaching, ward orientation, and community outreach, alongside supply-chain reforms. Health agencies and professional societies should integrate digital learning into national nursing curricula so hospital and community staff retain core FI-management skills.

Outside the hospital, care processes, staffing ratios, and reimbursement patterns differ markedly in long-term care facilities (LTCFs) and home-care settings (31). In LTCFs, high resident-to-staff ratios and rigid toileting schedules may deter the adoption of labor-intensive diversion devices, whereas constrained budgets still favor low-cost absorbent pads (31, 32). Home caregivers often confront limited product availability and rely on informal skills, making our findings on structured education and low-cost improvisation especially relevant (33). Nonetheless, all settings share common risks—frailty, prolonged stool exposure, and IAD—so evidence-based skin-care protocols and multidisciplinary collaboration are broadly applicable (34). Future stratified, multisite studies spanning hospitals, LTCFs, and home care are warranted to refine these observations.

Additionally, improvements to the supply chain are needed to ensure a broader range of appropriate product availability, helping nurses and caregivers make more informed decisions. Collaboration among healthcare professionals—including nurses, clinicians, and nutritionists—is essential for improving patient outcomes, particularly in preventing complications like IAD (35, 36). This also requires support from public health policies. By relying on these policies to facilitate multidisciplinary collaboration, we can promote teamwork among various disciplines, achieve early detection of IAD, and implement systematic training for advanced FI management.

This study provides valuable insights into the practical challenges faced by caregivers in managing FI and emphasizes the importance of product functionality and availability. At the public health level, enhancing the product supply chain and training systems can reduce the transmission of infections and alleviate the burden on the healthcare system, ultimately improving patient care quality and increasing the overall operational efficiency of healthcare institutions (37). By addressing these challenges, this study aims to contribute to better public health strategies that ultimately reduce HAIs, improve patient outcomes, and ease the burden on the healthcare system (6).

We employed a sequential explanatory mixed methods approach underpinned by pragmatism, integrating quantitative and qualitative data to gain a holistic understanding of the complex FI care environment (38, 39). This design enabled us to use quantitative surveys to map out the overall caregiving landscape and then deepen our understanding of underlying factors through qualitative interviews—enhancing both the conceptual and practical significance of the study.

### 4.1 Limitations

Although this study integrates quantitative and qualitative methods to provide a comprehensive understanding, it has several limitations. First, the relatively small sample sizes in certain subgroups (e.g., only 73 nursing assistants and 10 with college degrees) may limit the statistical robustness of some findings. Second, the study was conducted in three hospitals in southern China, potentially limiting generalizability to other regions or countries where long-term care might occur in nursing homes rather than in hospital settings. Third, qualitative participants were partially identified with administrative assistance, which may introduce selection bias. Fourth, we did not specifically measure HAIs due to participants' limited awareness of infection occurrence. Fifth, although our sample included one specialized ostomy nurse whose advanced expertise may introduce a degree of bias, the overall diversity of participants still offers broadly applicable insights for the general nursing population. Finally, we did not include perspectives from clinicians, administrators, or nutritionists, which could have enriched the multidisciplinary aspect of FI management. Larger-scale studies with more diverse stakeholder involvement are recommended to confirm and extend these findings.

## 5 Conclusions

This study demonstrates that absorbent products continue to dominate the management of fecal incontinence in long-term hospital settings—even among patients with incontinence-associated dermatitis (IAD)—primarily because of financial constraints, limited awareness, and an inadequate supply of advanced devices. Trained caregivers—particularly nurses and nursing assistants with specialized experience—are more inclined to use alternative devices, yet these options remain underutilized. Public-health policy should therefore focus on increasing the availability of fecal-diversion systems, broadening insurance coverage, and expanding multidisciplinary training programs. A more robust product-supply chain and greater caregiver support could reduce healthcare-associated infections (HAIs), lower IAD incidence, and improve patient outcomes. Future research should evaluate “smart” FI-management systems and assess their feasibility in long-erm hospital care, paving the way for comprehensive strategies that address the clinical, financial, and emotional challenges of fecal incontinence.

## Data Availability

The raw data supporting the conclusions of this article will be made available by the authors, without undue reservation.
